# Relationship Between Age and Pathology With Treatment of Pediatric and Adolescent Discoid Lateral Meniscus: A Report From the SCORE Multicenter Database

**DOI:** 10.1177/03635465231206173

**Published:** 2023-10-29

**Authors:** Members of the SCORE Quality Improvement Registry, Elizabeth Adsit, Jay Albright, Sheila Algan, Jennifer Beck, Richard E. Bowen, Jennifer Brey, J. Marc Cardelia, Christian Clark, Pablo Coello, Allison Crepeau, Eric Edmonds, Matthew Ellington, Henry B. Ellis, Peter D. Fabricant, Jeremy S. Frank, Theodore J. Ganley, Daniel W. Green, Andrew Gupta, Benton Heyworth, W. Craig Kemper, Kevin Latz, Alfred Mansour, Stephanie Mayer, Scott D. McKay, Matthew D. Milewski, Emily Niu, Donna M. Pacicca, Shital N. Parikh, Lauren Pupa, Jason Rhodes, Michael Saper, Gregory A. Schmale, Matthew Schmitz, Kevin Shea, Rachel S. Silverstein, Stephen Storer, Philip L. Wilson

**Affiliations:** Scottish Rite for Children, Dallas, Texas, USA; Department of Orthopedics, Children's Hospital Colorado, Aurora, Colorado, USA; Department of Orthopedic Surgery, Oklahoma Children's Hospital, Oklahoma City, Oklahoma, USA; Department of Orthopaedic Surgery, David Geffen School of Medicine at UCLA, Los Angeles, California, USA; Orthopedic Institute for Children's Center for Sports Medicine, Los Angeles, California, USA; Department of Orthopedics, Norton Children's Orthopedics of Louisville, Louisville, Kentucky, USA; Department of Orthopedics and Sports Medicine, Children's Hospital of the King's Daughters, Norfolk, Virginia, USA; OrthoCarolina Pediatric Orthopaedic Center, Charlotte, North Carolina, USA); Baylor College of Medicine, Houston, Texas, USA; Elite Sports Medicine at Connecticut Children's, Hartford, Connecticut, USA; Division of Sports Medicine, Department of Orthopedics, UConn Health, Farmington, Connecticut, USA; Division of Orthopaedic Surgery, Rady Children's Hospital, San Diego, California, USA; Department of Orthopedics, Central Texas Pediatric Orthopedics, Austin, Texas, USA; Dell Medical School, University of Texas at Austin, Austin, Texas, USA; University of Texas Southwestern Medical Center, Dallas, Texas, USA; Scottish Rite for Children, Dallas, Texas, USA; Division of Pediatric Orthopaedic Surgery, Hospital for Special Surgery, New York, New York, USA; Weill Cornell Medical College, New York, New York; Division of Pediatric Orthopaedics and Spinal Deformities, Joe DiMaggio Children's Hospital, Hollywood, Florida, USA; Division of Orthopaedics, Children's Hospital of Philadelphia, Philadelphia, Pennsylvania, USA; Division of Pediatric Orthopaedic Surgery, Hospital for Special Surgery, New York, New York, USA; Division of Pediatric Orthopaedics and Spinal Deformities, Joe DiMaggio Children's Hospital, Hollywood, Florida, USA; Department of Orthopaedic Surgery, Boston Children's Hospital, Boston, Massachusetts, USA; University of Texas Southwestern Medical Center, Dallas, Texas, USA; Department of Orthopedics-Sports Medicine, Children's Mercy, Kansas City, Missouri, USA; Department of Orthopedic Surgery, UTHealth Houston, McGovern Medical School, Houston, Texas, USA; Department of Orthopedics, Children's Hospital Colorado, Aurora, Colorado, USA; Baylor College of Medicine, Houston, Texas, USA; Texas Children's Hospital, Houston, Texas, USA; Department of Orthopaedic Surgery, Boston Children's Hospital, Boston, Massachusetts, USA; Department of Orthopedic Surgery and Sports Medicine, Children's National Medical Center, Washington, DC, USA; Department of Orthopedics-Sports Medicine, Children's Mercy, Kansas City, Missouri, USA; Division of Orthopaedic Surgery, Cincinnati Children's Hospital Medical Center, Cincinnati, Ohio, USA; Baylor College of Medicine, Houston, Texas, USA; Department of Orthopedics, Children's Hospital Colorado, Aurora, Colorado, USA; Department of Orthopedics and Sports Medicine, Seattle Children's Hospital, Seattle, Washington, USA; San Antonio Military Medical Center, San Antonio, Texas, USA; Department of Orthopaedics, Stanford University School of Medicine, Stanford, California, USA; Baylor College of Medicine, Houston, Texas, USA; Texas Children's Hospital, Houston, Texas, USA; Division of Pediatric Orthopaedics and Spinal Deformities, Joe DiMaggio Children's Hospital, Hollywood, Florida, USA; University of Texas Southwestern Medical Center, Dallas, Texas, USA; Scottish Rite for Children, Dallas, Texas, USA); Investigation performed at Scottish Rite for Children, University of Texas Southwestern Medical Center, Dallas, USA

**Keywords:** discoid meniscus, meniscal tear, instability, pediatric, saucerization

## Abstract

**Background:**

Surgical treatment options of discoid lateral meniscus in pediatric patients consist of saucerization with or without meniscal repair, meniscocapular stabilization, and, less often, subtotal meniscectomy.

**Purpose:**

To describe a large, prospectively collected multicenter cohort of discoid menisci undergoing surgical intervention, and further investigate corresponding treatment of discoid menisci.

**Study Design:**

Cohort study; Level of evidence, 3.

**Methods:**

A multicenter quality improvement registry (16 institutions, 26 surgeons), Sports Cohort Outcomes Registry, was queried. Patient characteristics, discoid type, presence and type of intrasubstance meniscal tear, peripheral rim instability, repair technique, and partial meniscectomy/debridement beyond saucerization were reviewed. Discoid meniscus characteristics were compared between age groups (<14 and >14 years old), based on receiver operating characteristic curve, and discoid morphology (complete and incomplete).

**Results:**

In total, 274 patients were identified (mean age, 12.4 years; range, 3-18 years), of whom 55.6% had complete discoid. Meniscal repairs were performed in 55.1% of patients. Overall, 48.5% of patients had rim instability and 36.8% had >1 location of peripheral rim instability. Of the patients, 21.5% underwent meniscal debridement beyond saucerization, with 8.4% undergoing a subtotal meniscectomy. Patients <14 years of age were more likely to have a complete discoid meniscus (*P* < .001), peripheral rim instability (*P* = .005), and longitudinal tears (*P* = .015) and require a meniscal repair (*P* < .001). Patients ≥14 years of age were more likely to have a radial/oblique tear (*P* = .015) and require additional debridement beyond the physiologic rim (*P* = .003). Overall, 70% of patients <14 years of age were found to have a complete discoid meniscus necessitating saucerization, and >50% in this young age group required peripheral stabilization/repair.

**Conclusion:**

To preserve physiological “normal” meniscus, a repair may be indicated in >50% of patients <14 years of age but occurred in <50% of those >14 years. Additional resection beyond the physiological rim may be needed in 15% of younger patients and 30% of those aged >14 years.

Menisci are crescent-shaped, fibrocartilaginous structures found between the tibia and femur that act to disburse weight and reduce friction for the knee articular cartilage, assisting in static and dynamic stability.^24^ The discoid meniscus is an unusual anatomic variant with increased thickness, abnormal width and shape, poor vascularization, and compromised joint capsule attachments leading to peripheral rim instability.^5,17,37^ Lateral discoid meniscus is more common than medial discoid meniscus, with incidence rates in the United States of 1.5% to 5.2% and 0.06% to 3%, respectively.^12,19,33,39,43^

A discoid meniscus has several anatomic differences when compared with a normal meniscus that predispose individuals with this variant to injury.^11^ It has a thicker outer rim with greater surface area coverage on the tibial plateau.^5,8,26,27^ These differences alter its physiologic ability to transmit forces and result in more mechanical and shearing stresses placed on the meniscus.^16^ Additionally, it has a decreased peripheral vascular supply, and thus less ability to repair itself.^7^ Lastly, histologic evaluation of discoid meniscus has shown decreased numbers of type 1 collagen fibers and decreased organization of fibers compared with normal menisci.^5,7,20,27,33^ The discoid meniscus has abnormalities of both internal microscopic structures and gross morphology. These variations from normal anatomy increase susceptibility to tears at an earlier age.^16,25,27^

Historically, treatments of symptomatic discoid meniscus, including total or subtotal meniscectomy, led to the development of early disability and degenerative changes.^2,9,15,28,35^ More recently, strategies in the treatment of a discoid meniscus focus on meniscal preservation.^2,9,35,45^ One such technique is saucerization with or without repair of a meniscal intrasubstance tear and/or torn or unstable meniscocapsular attachments.^1-3,9,23,25,30,34,35,45^ Addressing abnormal discoid meniscus tissue at an earlier age may avoid the need for a subtotal meniscectomy or the development of osteochondral changes to the lateral compartment. The goal is to reshape the meniscus by excising the central abnormal tissue to a stable 6- to 8-mm rim to resemble a more normal crescent-shaped anatomy.^1,2,5,17,35,36,45^ In discoid treatment, preservation of more meniscal tissue may lessen the tendency to develop degenerative changes.^45^

The treatment of discoid meniscus in a symptomatic patient is individualized based on the presentation, discoid morphologic type, and presence of an intrasubstance tear and/or rim instability. As potential treatment options and requirements may vary, the surgeon needs to be prepared for the appropriate intervention. It has been found that age is an important prognostic factor in clinical outcomes.^26^ Therefore, the purpose of this study was to describe a large, prospectively collected multicenter cohort of discoid menisci requiring surgical intervention and further investigate the corresponding treatment of discoid menisci. Secondarily, we evaluated whether age influences treatment, including meniscal repair or meniscal resection.

## Methods

This is a retrospective review of prospectively collected data from the Sports Cohort Outcomes Registry (SCORE). This is a surgeon-driven quality improvement initiative that includes 26 pediatric and/or sports medicine fellowship-trained surgeons from 16 institutions. Participating surgeons are required to enter consecutive patients <18 years of age undergoing surgical treatment for a discoid meniscus.

Institutional review board approval or exemption was obtained at the decision and discretion of each institution for the SCORE quality improvement registry. Data for the current report included cases occurring between July 15, 2018, and December 1, 2020. Data were de-identified for analysis by the host institution. Data quality in the registry was maintained using a biannual audit ensuring that each surgeon and institution entered consecutive patients into the registry. The electronic database is a Health Insurance Portability and Accountability Act (HIPAA)-compliant platform (Scribe System; Web Data Solutions) in which the data extraction included no identifiable data.

Surgical treatment for a lateral discoid meniscus, either partial debridement (saucerization) and/or repair, was queried from the registry as a categorical variable. Nonsurgical management of discoid meniscus was not included in the registry. Indications for treatment of a discoid meniscus were determined by the participating surgeon.

Surgeons were required to enter data within 2 weeks of the surgery and strongly encouraged to enter data within 24 hours of the procedure to avoid recall bias. Patient data, including age, sex, date of injury, and postoperative management, were included. After the surgery, surgeons entered the morphologic type of discoid meniscus according to the Watanabe classification^42^ (complete, incomplete, or Wrisberg), as well as the presence and location of peripheral rim instability (anterior, posterior, body, or a combination of locations). Peripheral rim meniscal pathology possibly due to either an acute or chronic meniscocapsular junction tear or an absent peripheral connection was defined in this study as rim instability. If a meniscal intrasubstance tear was present, the surgeon noted the type of meniscal tear (complex/degenerative, horizontal/cleavage, longitudinal/vertical, oblique/parrot beak, or radial) and location of displacement. Surgeons were also required to document treatment, including saucerization and whether during a saucerization meniscal resection was performed beyond a normal physiological meniscal rim (6-8 mm; based on size and age of the patient as determined by the surgeon). Surgeons were asked to provide a percentage of additional rim resection beyond a normal physiologic rim resection and the location of resection. If >50% of resection was required in >1 location on the meniscus or 100% in any 1 location, the resection was considered a subtotal meniscectomy. Location (anterior, body, posterior, subtotal, or combination of locations) and percentage of additional meniscal resection were also reported in the registry to determine when the morphology of the discoid warranted additional resection. Finally, if a repair was performed, it was documented if it was for an intrasubstance meniscal tear, rim instability, or both.

Patients were excluded if incomplete data were available in the database, if they had a Wrisberg variant of discoid meniscus, or if the patients were ≥18 years of age.

### Statistical Analysis

Discoid meniscus data were organized to present a large set of epidemiologic descriptive analysis. Patients were classified by age groups of either <14 years or ≥14 years. This was determined by constructing a receiver operating characteristic (ROC) curve analysis to identify a threshold value, which was determined to be age <14 years for type of discoid, need for repair, and saucerization. Age groups were also broken down into <7, 7 to 10, 11 to 13, 14 to 16, and >16 years old based on a previous publication by Ellis et al.^11^ The data were also analyzed to find differences in meniscal pathology and treatment based on whether the discoid meniscus was complete or incomplete.

Continuous variables were first examined for normality, and a nonparametric test such as the Kruskal-Wallis test was considered. For multiple comparisons, the Tukey-Kramer method and the nonparametric method by Dwass, Steel, and Critchlow-Fligner were used as appropriate. A chi-square test was used for categorical variables, and for small sample, a Fisher exact test was used. *P* < .05 was considered statistically significant. Statistical analyses were performed by SPSS software (Version 27; IBM Corp) and the ROC package (Version 4.0.1; R Development Core Team; www.r-project.org).

## Results

In total, 274 consecutive patients underwent operative intervention for their symptomatic lateral discoid meniscus pathology. The mean age was 12.4 years (range, 3-18 years). There were 142 (52.4%) male patients and 129 (47.6%) female patients. Complete discoid morphology was found in 156 (57.6%) patients, and incomplete discoid morphology was found in 115 (42.4%) patients (Table 1). Peripheral instability was noted in 49.1% of surgically treated discoid menisci, with a majority in >1 location (36.8%). Rim instability in a combination of locations in 49 patients was found to be 26.5% anterior and body; 28.6% anterior and posterior; 8.2% anterior, body, and posterior; and 36.7% posterior and body. Meniscal intrasubstance tears were common in 80.1% of discoid menisci, with the most common tear being the complex/degenerative tear (46.5%). During saucerization, 23 (8.4%) patients underwent subtotal meniscectomy in any one location, while an additional 20 (7.3%) underwent a meniscal resection of >50% in any one location (see Table 3). Meniscal repair was performed for an isolated intrasubstance meniscal tear in 36 (24.3%) patients, isolated rim instability was repaired in 13 (8.8%) patients, and a combination of intrasubstance meniscal tear and rim instability was repaired in 99 (66.9%) patients (see Table 2).

**Table 1:** Meniscal Pathology in the Entire Cohort With Comparison Between the <14- and ≥14-Year Age Groups^*a*^

Meniscal Pathology	Total (N = 271)	<14 Y (n = 153)	≥14 Y (n = 118)	*P* Value
Discoid type				**<.001**
Complete	156 (57.6)	107 (69.9)	49 (41.5)	
Incomplete	115 (42.4)	46 (30.1)	69 (58.5)	
Rim instability				**.004**
Yes	133 (49.1)	87 (56.9)	46 (39.0)	
No	138 (50.9)	66 (43.1)	72 (61.0)	
Rim instability location	n = 133	n = 87	n = 46	**.015**
Anterior	37 (27.8)	23 (26.4)	14 (30.4)	
Posterior	38 (28.6)	29 (33.3)	9 (19.6)	
Body	9 (6.8)	3 (3.4)	6 (13.0)	
Combination	49 (36.8)	32 (36.8)	17 (37.0)	
Presence of intrasubstance tear				.281
Yes	217 (80.1)	119 (77.8)	98 (83.1)	
No	54 (19.9)	34 (22.2)	20 (16.9)	
Type of tear	n = 217	n = 119	n = 98	**.002**
Complex/degenerative	101 (46.5)	56 (47.1)	45 (45.9)	
Horizontal/cleavage	45 (20.7)	25 (21.0)	20 (20.4)	
Longitudinal/vertical	38 (17.5)	29 (24.4)	9 (9.2)	
Oblique/parrot beak	13 (6.0)	3 (2.5)	10 (10.2)	
Radial	20 (9.2)	6 (5.0)	14 (14.3)	

a Data are presented as n (%). Boldface *P* values indicate statistical significance.

**Table 2:** Treatment in the Entire Cohort With Comparison Between the <14- and ≥14-Year Age Groups^*a*^

Treatment	Total (n = 271)	<14 Y (n = 153)	≥14 Y (n = 118)	*P* Value
Saucerization				**.005**
Yes	254 (93.7)	149 (97.4)	105 (89.0)	
No	17 (6.3)	4 (2.6)	13 (11.9)	
Additional debridement				**.004**
Yes	60 (22.1)	24 (15.7)	36 (30.5)	
No	211 (77.9)	129 (84.3)	82 (69.5)	
Additional debridement location	n = 60	n = 24	n = 36	**.004**
Anterior	3 (5.0)	1 (4.2)	2 (5.6)	
Body	21 (35.0)	2 (8.3)	19 (52.8)	
Posterior	2 (3.3)	2 (8.3)	00 (0.0)	
Subtotal	4 (6.7)	3 (12.5)	1 (2.8)	
Combination	30 (50.0)	16 (66.7)	14 (38.9)	
Repair		n = 153	n = 118	**.002**
Yes	148 (54.6)	96 (62.7)	52 (44.1)	
No	123 (45.4)	57 (37.3)	66 (55.9)	
Repair type	n = 148	n = 96	n = 52	**.017**
Intrasubstance tear	36 (24.3)	18 (18.8)	18 (34.6)	
Rim instability	13 (8.8)	6 (6.3)	7 (13.5)	
Combination	99 (66.9)	72 (75.0)	27 (51.9)	

a Data are presented as n (%). Boldface *P* values indicate statistical significance.

### Age: <14 Years Versus ≥14 Years

A total of 153 (56.5%) patients were <14 years of age and 118 (43.5%) patients were ≥14 years of age. In the <14-year group, the sexes were more evenly distributed, with 69 (45.1%) male patients and 84 (54.9%) female patients. However, in the ≥14-year group there were considerably more male patients than female patients (73 [61.9%] vs 45 [38.1%]; *P* = .006). Complete discoid meniscus was more common in the <14-year group (69.9%) of patients, while incomplete discoid meniscus was more common in the ≥14-year group (58.5%) (*P* < .001) (Table 1).

Rim instability was found in 133 (49.1%) patients: 87 (65.4%) patients in the <14-year group and 46 (34.6%) patients in the ≥14-year group. Difference in the rate of rim instability between the <14-year and ≥14-year groups was statistically significant, with rim instability occurring in 56.9% of the <14-year group but only 39.0% of the ≥14-year group (*P* = .004). For both age groups, the location of rim instability was most commonly multifocal or combined location (posterior and body being the most common combination at 36.8%). Posterior rim instability was more common in the <14-year group (*P* = .015).

Meniscal intrasubstance tears were found in 217 (80.1%) patients and were equally common in both age groups (54.8% in the ≥14-year group and 45.2% in the <14-year group). Complex and horizontal tears made up two-thirds of both groups’ tears. There were more longitudinal tears in the <14-year group (24.4% vs 9.2%) and more radial and oblique tears in the ≥14-year group (24.5% vs 7.5%). Overall, the morphology of the meniscal tears was different between age groups (*P* = .002).

Arthroscopic partial meniscectomy in the form of saucerization of the discoid meniscus was performed in 254 (93.7%) patients (Table 2). Saucerization was performed more frequently in the <14-year (97.4%) versus the ≥14-year (89.0%) group (*P* = .005). Additional debridement rates between age groups were significantly different, with additional debridement performed in 15.7% of <14-year patients compared with 30.5% of ≥14-year patients (*P* = .004). Multifocal locations of additional debridement were most common in the <14-year group at 66.7% versus 38.9% in the ≥14-year group (*P* = .004). The body of the meniscus was the most common location of additional debridement for the ≥14-year group at 52.8% compared with only 8.3% in the <14-year group (*P* = .004). Between 20-50% of additional meniscus was debrided during saucerization most commonly in the body for 26 patients (10.2%), while 20 (7.8%) underwent a meniscal resection of >50% in any 1 location. 23 (9.1%) patients underwent a subtotal meniscectomy alone or in combination with another debridement location (Table 3).

**Table 3:** Resection of Discoid Meniscus Beyond a Physiologic Rim (6-8 mm)^*a*^

Location of	Percentage of Meniscal Resection (n = 254)		
Meniscal Resection	<20%	20%-50%	>50%
Anterior	1 (0.4)	9 (3.5)	1 (0.4)
Body	1 (0.4)	26 (10.2)	4 (1.6)
Posterior		9 (3.5)	1 (0.4)
Subtotal			23 (9.1)

a Data are presented as n (%).

Meniscal repair was performed in 148 (54.6%) patients. Meniscal repair occurred more frequently in the <14-year group (62.7%) compared with only 44.1% in the ≥14-year group (*P* = .002) (Table 2). In the <14-year group, there was a higher percentage of repair of both intrasubstance meniscal tears and rim instability at 75.0% compared with 51.9% in the ≥14-year group (*P* = 0.17).

### Age Groups

**Figure 1.**
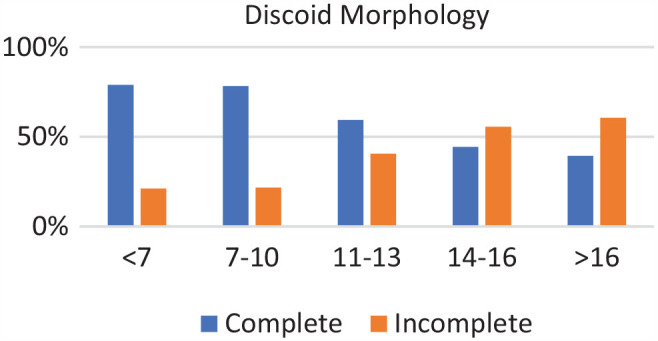
Discoid morphology by age groups. Incidence of complete discoid morphology decreased with age while incomplete morphology increased with age.

**Figure 2.**
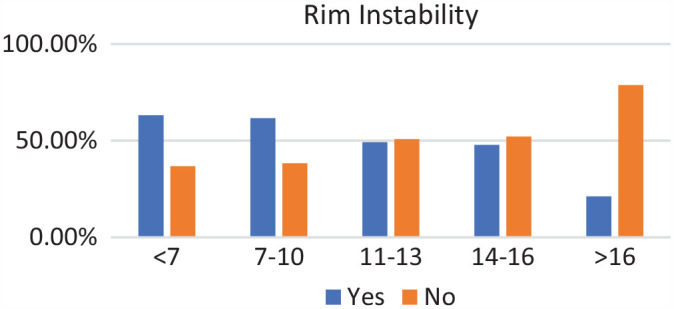
Rim instability by age groups. Incidence of rim instability decreased with age.

**Figure 3.**
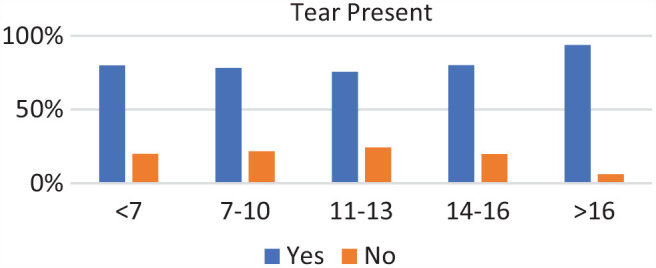
Tear present by age groups. Incidence of a tear was common among all age groups.

**Figure 4.**
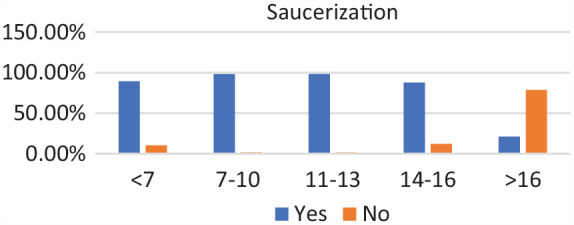
Saucerization by age groups. Saucerization remained stable with a sharp drop-off after age 14 to 16 years.

**Figure 5.**
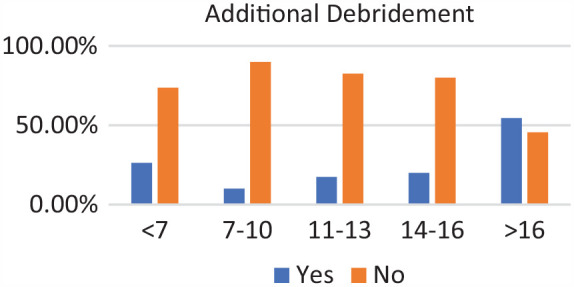
Additional debridement by age groups. Incidence of additional debridement increased with age.

**Figure 6.**
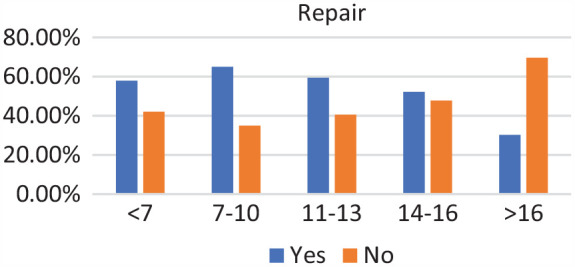
Repair by age groups (for rim instability, tear, or both). Incidence of repair decreased with age.

Of the 271 patients, 19 were in the <7-year age group, 61 were in the 7- to 10-year age group, 73 were in the 11- to 13-year age group, 89 were in the 14- to 16-year age group, and 29 were in >16-year age group. Complete discoid was more common in the <7-year, 7- to 10-year, and 11- to 13-year age groups, and incomplete discoid morphology was more common in the 14- to 16-year and >16-year age groups (*P* < .001) (Figure 1). Rim instability was found more often in <7- and 7- to 10-year age groups and rarely found in the >16-year age group (*P* < .001) (Figure 2). No differences were found in the incidence of intrasubstance meniscal tears among all age groups (Figure 3). Saucerization of the discoid meniscus was performed in the <7-year, 7- to 10-year, 11- to 13-year, and 14- to 16-year age groups and rarely performed in the >16-year age group (*P* < .001) (Figure 4). Additional debridement beyond physiological rim was performed in the >16-year old age group and less common in the <7-year, 7- to 10-year, 11- to 13-year, and 14- to 16-year age groups (*P* < .002) (Figure 5). Meniscal repair occurred more frequently in the <7-year, 7- to 10-year, and 11- to 13-year age groups and less commonly in the >16-year old age group (*P* = .025) (Figure 6).

### Discoid Morphology

Complete discoid menisci were more likely to have rim instability than incomplete discoid meniscus (59.6% vs 34.8%; *P* < .001) (Table 4). Intrasubstance tear type distribution when comparing complete and incomplete discoid meniscus subcohorts was significantly different (*P* < .001). Nearly half of each group's tears were complex, but there were more radial and parrot beak tears in the incomplete group (29.5%) than in the complete group (5.3%).

**Table 4:** Meniscal Pathology and Treatment in the Complete and Incomplete Discoid Meniscus Groups^*a*^

	Complete Discoid (n = 156)	Incomplete Discoid (n = 115)	*P* Value
Meniscal pathology			
Rim instability			**<.001**
Yes	93 (59.6)	40 (34.8)	
No	63 (40.4)	75 (65.2)	
Rim instability location	n = 93	n = 40	.056
Anterior	22 (23.7)	15 (37.5)	
Posterior	25 (26.9)	13 (32.5)	
Body	5 (5.4)	4 (10.0)	
Combination	41 (44.1)	8 (20.0)	
Presence of intrasubstance tear			.167
Yes	130 (83.3)	88 (76.5)	
No	26 (16.7)	27 (23.5)	
Type of tear	n = 130	n = 88	**<.001**
Complex/degenerative	62 (47.7)	39 (44.3)	
Horizontal/cleavage	36 (27.7)	10 (11.4)	
Longitudinal/vertical	25 (19.2)	13 (14.8)	
Oblique/parrot beak	2 (1.5)	11 (12.5)	
Radial	5 (3.8)	15 (17.0)	
Treatment			
Saucerization			.184
Yes	150 (96.2)	106 (92.2)	
No	6 (3.8)	9 (7.8)	
Additional debridement			.457
Yes	31 (19.9)	28 (24.3)	
No	125 (80.1)	87 (75.7)	
Additional debridement location	n = 31	n = 28	.102
Anterior	2 (6.5)	1 (3.6)	
Body	6 (19.3)	15 (53.6)	
Posterior	1 (3.2)	1 (3.6)	
Subtotal	2 (6.5)	1 (3.6)	
Combination	20 (64.5)	10 (35.7)	
Repair			**.002**
Yes	98 (62.8)	50 (43.5)	
No	58 (37.2)	65 (56.5)	
Repair type	n = 98	n = 50	**.021**
Intrasubstance tear	17 (17.3)	19 (38.0)	
Rim instability	9 (9.2)	4 (8.0)	
Combination	72 (73.5)	27 (54.0)	

a Data are presented as n (%). Boldface *P* values indicate statistical significance.

Meniscal repair was more likely to be performed in complete discoid meniscus cases versus incomplete cases (62.8% vs 43.5%; *P* = .002). The complete discoid group was more likely to require repair for a combination of rim instability and intrasubstance tear (74.5% vs 52.8%; *P* = .021). Additional debridement beyond the physiologic rim was performed in both complete and incomplete discoid groups, but still in a minority of cases (19.9% and 24.3%). This was not statistically significant (Table 4).

## Discussion

The most important findings in our study were the high rates of peripheral rim instability, meniscal repairs, and subtotal meniscectomies required compared with the rates reported in the existing literature. Younger patients had more complete discoid variants, were more likely to have rim instability, and were more likely to require meniscal repair for any reason. Older patients had more incomplete discoid menisci and additional debridement beyond the physiologic rim. Degenerative/complex tears were most common overall, but the younger patients were more likely to have longitudinal tears, and the older group was more likely to have radial and oblique tears. Complete discoid menisci more frequently demonstrated rim instability and were therefore more likely to require meniscal repair for any reason. Discoid menisci that required debridement beyond the physiologic rim were equally uncommon in both discoid variants.

Peripheral rim instability occurs due to acute or chronic meniscocapsular junction tears or abnormal, attenuated, or absent meniscocapsular attachments of the discoid meniscus and is postulated to be in areas of higher stress.^3,21,25^ If the rim can be everted or translated to the other half of the tibial plateau after saucerization, it is considered unstable.^14^ The incidence of rim instability in pediatric patients has variable rates in the existing literature, from 28% to 77%.^3,14,31,37^ The findings of the present study indicated rim instability in 49% of patients. We also found a higher percentage in the younger age group, and more commonly in complete discoid meniscus variants, which aligns with the current literature.^11,14,27^ Rim instability classification was first described by Klingele et al^23^ as stable or unstable, torn or intact rim, and complete or incomplete discoid variants. Good et al^14^ further categorized by location. Rim instability in our study was further categorized into anterior, body, posterior horn, or multifocal instability. Most of our patients had multifocal instability, with body and posterior combination the most common, while isolated anterior or posterior rim instability occurred with similar frequency. However, we did find a slightly higher frequency of anterior instability in the older age group versus more posterior instability in the younger age group. The previous literature varies, with some finding anterior instability to be more common and vice versa.^11,14,23,34,38,44^

Discoid menisci have been found to have a higher frequency of intrasubstance tears than normal meniscal morphology, ranging in the current literature from 33.8% to 5%.^†^ Many factors may contribute to this, such as an increased thickness leading to abnormal shearing forces between the layers,^14,27^ as well as microstructural instability due to disorganized collagen fibers.^3,5,10,14,18,40^ In our study, we found intrasubstance tears in 80% of discoid patients. Intrasubstance tears were slightly more prevalent in complete (83.3%) than incomplete (76.5%) variants, which is consistent with the literature.^14,36^

There is no consensus with regard to which intrasubstance meniscal tear pattern is the most common, or if there is a correlation to meniscal type and age. Some studies have reported that the most common tear pattern of the discoid meniscus is horizontal cleavage^3,5,9,14,29,36,40,44^; others have found longitudinal tears to be the most common,^4,17,18,26^ and still others have found complex tears to be the most common.^13,28^ In this cohort, complex tears were the most common (47%). Younger patients had a higher percentage of longitudinal tears, and older patients had a higher percentage of oblique and radial tears. One reason that tear patterns may differ between studies is differences in age inclusion criteria.

Horizontal tears are the most frequent type of tear pattern reported in complete discoid meniscus^3,5,6,14,30,31,36^; however, some report vertical/longitudinal tears as most common.^1,4,17,22,32,41^ We found that complex tear patterns were the most common for complete discoid variants; however, horizonal and longitudinal tears were more common than in incomplete variants. Complex tears were also the most common for incomplete discoid variants, but oblique and radial tears were more common than complete variants. This is similar to current literature, in which studies found radial,^5,6,22^ longitudinal,^4,6,26,41^ or complex^6^ tears to be the most common for incomplete discoid variants. In any case, the operating surgeon needs to be aware that the abnormal meniscus shape and structure seem to predispose the discoid meniscus to intrasubstance meniscal tears of any type, and that surgical treatment may require expertise with multiple stabilization techniques.

Saucerization is reshaping the discoid meniscus by resecting the central portion of abnormal tissue to restore its mechanical function and creating a stable peripheral rim^1,27^ to provide shock absorption while minimizing the risk of retear.^1,5,43^ In our study, 94% of patients with discoid meniscus underwent saucerization. Younger patients and those with complete discoid variants underwent saucerization more frequently. Older patients with incomplete discoid morphology had the lowest rate of saucerization. There is no literature assessing differences in the need for saucerization based on age and discoid variant. After arthroscopic partial meniscectomy with saucerization, if the remaining meniscal morphology is poor, then additional debridement may be needed. In our study, additional debridement was performed in 22% of all patients regardless of age. It was necessary in up to 61% of the older age group.

Meniscal repair for the discoid meniscus has been found to be between 18% and 46% in the literature.^2,3,8,11,30,31,38^ Our study population had a repair rate of 55%. We also found that repair was more often performed for younger patients and complete discoid variants. This is in accordance with literature that found that repair was higher in younger patients.^11^ The most common repair technique was all-inside repair for all age groups and both complete and incomplete discoid morphology types.

Limitations to our study include the use of a quality improvement registry for the purposes of descriptive epidemiologic study on discoid meniscus, as the primary intention of the registry is to report complications associated with the operative treatment of discoid menisci, and not specifically to describe discoid meniscus correlates. Contributing surgeons likely have variability in the classification and description as well as operative indications and treatment. Reliability in the classification and intraoperative description of the discoid meniscus was not confirmed. There also might be recall bias as surgeons had up to 2 weeks to input information into the database.

## Conclusion

Data on operatively treated discoid menisci in pediatric and adolescent patients recorded in the SCORE database reveal key differences in age, sex, and discoid variant type. Higher rates of peripheral rim instability, meniscal repair, stabilization procedures, and subtotal meniscectomies than previously reported were noted.

This investigation identified important information for surgeon preparation and family counseling. Surgeons should be aware that 70% of patients <14 years of age will have a complete discoid meniscus requiring saucerization, and slightly more than 50% in this age group will require peripheral stabilization/repair. Patient and family counseling should include the understanding that meniscal repair may be required in >50% of patients <14 years of age but not in those ≥14 years of age. Furthermore, additional resection, resulting in significant meniscal functional loss, may be required in 15% of younger patients and 30% of those aged ≥14 years. Future research is still needed on this topic to address ideal treatment strategies and long-term outcomes.

## Authors

*Members of the SCORE Quality Improvement Registry*: Elizabeth Adsit (Scottish Rite for Children, Dallas, Texas, USA); Jay Albright, MD (Department of Orthopedics, Children's Hospital Colorado, Aurora, Colorado, USA); Sheila Algan, MD (Department of Orthopedic Surgery, Oklahoma Children's Hospital, Oklahoma City, Oklahoma, USA); Jennifer Beck, MD, Richard E. Bowen, MD (Department of Orthopaedic Surgery, David Geffen School of Medicine at UCLA, Los Angeles, California, USA; Orthopedic Institute for Children's Center for Sports Medicine, Los Angeles, California, USA); Jennifer Brey, MD (Department of Orthopedics, Norton Children's Orthopedics of Louisville, Louisville, Kentucky, USA); J. Marc Cardelia, MD (Department of Orthopedics and Sports Medicine, Children's Hospital of the King's Daughters, Norfolk, Virginia, USA); Christian Clark, MD (OrthoCarolina Pediatric Orthopaedic Center, Charlotte, North Carolina, USA); Pablo Coello, BS (Baylor College of Medicine, Houston, Texas, USA); Allison Crepeau, MD (Elite Sports Medicine at Connecticut Children's, Hartford, Connecticut, USA; Division of Sports Medicine, Department of Orthopedics, UConn Health, Farmington, Connecticut, USA); Eric Edmonds, MD (Division of Orthopaedic Surgery, Rady Children's Hospital, San Diego, California, USA); Matthew Ellington, MD (Department of Orthopedics, Central Texas Pediatric Orthopedics, Austin, Texas, USA; Dell Medical School, University of Texas at Austin, Austin, Texas, USA); Henry B. Ellis Jr., MD (University of Texas Southwestern Medical Center, Dallas, Texas, USA; Scottish Rite for Children, Dallas, Texas, USA); Peter D. Fabricant, MD (Division of Pediatric Orthopaedic Surgery, Hospital for Special Surgery, New York, New York, USA; Weill Cornell Medical College, New York, New York); Jeremy S. Frank, MD (Division of Pediatric Orthopaedics and Spinal Deformities, Joe DiMaggio Children's Hospital, Hollywood, Florida, USA); Theodore J. Ganley, MD (Division of Orthopaedics, Children's Hospital of Philadelphia, Philadelphia, Pennsylvania, USA); Daniel W. Green, MD (Division of Pediatric Orthopaedic Surgery, Hospital for Special Surgery, New York, New York, USA); Andrew Gupta, MD (Division of Pediatric Orthopaedics and Spinal Deformities, Joe DiMaggio Children's Hospital, Hollywood, Florida, USA); Benton Heyworth, MD (Department of Orthopaedic Surgery, Boston Children's Hospital, Boston, Massachusetts, USA); W. Craig Kemper, MD (University of Texas Southwestern Medical Center, Dallas, Texas, USA); Kevin Latz, MD (Department of Orthopedics-Sports Medicine, Children's Mercy, Kansas City, Missouri, USA); Alfred Mansour, MD (Department of Orthopedic Surgery, UTHealth Houston, McGovern Medical School, Houston, Texas, USA); Stephanie Mayer, MD (Department of Orthopedics, Children's Hospital Colorado, Aurora, Colorado, USA); Scott D. McKay, MD (Baylor College of Medicine, Houston, Texas, USA; Texas Children's Hospital, Houston, Texas, USA); Matthew D. Milewski, MD (Department of Orthopaedic Surgery, Boston Children's Hospital, Boston, Massachusetts, USA); Emily Niu, MD (Department of Orthopedic Surgery and Sports Medicine, Children's National Medical Center, Washington, DC, USA); Donna M. Pacicca, MD (Department of Orthopedics-Sports Medicine, Children's Mercy, Kansas City, Missouri, USA); Shital N. Parikh, MD (Division of Orthopaedic Surgery, Cincinnati Children's Hospital Medical Center, Cincinnati, Ohio, USA); Lauren Pupa, BA (Baylor College of Medicine, Houston, Texas, USA); Jason Rhodes, MD (Department of Orthopedics, Children's Hospital Colorado, Aurora, Colorado, USA); Michael Saper, MD, Gregory A. Schmale, MD (Department of Orthopedics and Sports Medicine, Seattle Children's Hospital, Seattle, Washington, USA); Matthew Schmitz, MD (San Antonio Military Medical Center, San Antonio, Texas, USA); Kevin Shea, MD (Department of Orthopaedics, Stanford University School of Medicine, Stanford, California, USA); Rachel S. Silverstein, MD (Baylor College of Medicine, Houston, Texas, USA; Texas Children's Hospital, Houston, Texas, USA); Stephen Storer, MD (Division of Pediatric Orthopaedics and Spinal Deformities, Joe DiMaggio Children's Hospital, Hollywood, Florida, USA); and Philip L. Wilson, MD (University of Texas Southwestern Medical Center, Dallas, Texas, USA; Scottish Rite for Children, Dallas, Texas, USA).

## Supplemental Material

sj-pdf-1-ajs-10.1177_03635465231206173 – Supplemental material for Relationship Between Age and Pathology With Treatment of Pediatric and Adolescent Discoid Lateral Meniscus: A Report From the SCORE Multicenter Database Click here for additional data file. Supplemental material, sj-pdf-1-ajs-10.1177_03635465231206173 for Relationship Between Age and Pathology With Treatment of Pediatric and Adolescent Discoid Lateral Meniscus: A Report From the SCORE Multicenter Database by Elizabeth Adsit, Jay Albright, Sheila Algan, Jennifer Beck, Richard E. Bowen, Jennifer Brey, J. Marc Cardelia, Christian Clark, Allison Crepeau, Eric Edmonds, Matthew Ellington, Henry B. Ellis, Peter D. Fabricant, Jeremy S. Frank, Theodore J. Ganley, Daniel W. Green, Andrew Gupta, Benton Heyworth, W. Craig Kemper, Kevin Latz, Alfred Mansour, Stephanie Mayer, Scott D. McKay, Matthew D. Milewski, Emily Niu, Donna M. Pacicca, Shital N. Parikh, Lauren Pupa, Jason Rhodes, Michael Saper, Gregory A. Schmale, Matthew Schmitz, Kevin Shea, Rachel S. Silverstein, Stephen Storer and Philip L. Wilson in The American Journal of Sports Medicine

## Footnotes

† References 3, 4, 6, 14, 21-23, 26, 32, 36-39, 44.
