# Supplementary material for: Relationship Between Age and Pathology With Treatment of Pediatric and Adolescent Discoid Lateral Meniscus: A Report From the SCORE Multicenter Database
Source: Am J Sports Med. 2023 Oct 29;51(13):3493–501. doi: 10.1177/03635465231206173 (PMC10623608; doi:10.1177/03635465231206173)
Supplement: sj-pdf-1-ajs-10.1177_03635465231206173 – Supplemental material for Relationship Between Age and Pathology With Treatment of Pediatric and Adolescent Discoid Lateral Meniscus: A Report From the SCORE Multicenter Database [file sj-pdf-1-ajs-10.1177_03635465231206173.pdf]

**Author Disclosure Statement:**

In the normal course of the conduct and publication of medical research, associations commonly exist that might prevent an author from reporting research with complete objectivity, or that might be perceived as doing so. The AJSM has adopted a policy of "transparency" in such matters, so that any circumstance that might be perceived as interfering with objectivity is openly declared. Individuals should note that a "potential conflict of interest" describes a circumstance or association, not a behavior, and does not imply that one's behavior has actually been influenced by the potentially conflicting circumstance.

<u>Each author should answer the following 5 questions directly related to the submitted work:</u>

1. Have you received any funding or sponsorship for this study, including funds or materials (e.g., surgical supplies, specimens)?

2. Have you, in the past five years, received the following from an organization that may in any way gain or lose financially from the results of your study:

- Reimbursement for attending a symposium?
- A fee for speaking or for organizing an educational program?
- Payment to write or review a paper?
- Funds for research?
- Funds for one of your employees?
- Royalties or fees for consulting?

3. Have you, in the past five years, been employed by or on the Board of an organization that may in any way gain or lose financially from the results of your study?

4. Do you hold any stocks or bonds in an organization that may in any way gain or lose financially from the results of your study? (This excludes stocks or bonds that may be held in mutual funds.)

5. Do you, or any entity with which you are affiliated, currently hold any patents that are relevant to the study in any way, or are you currently applying for or planning to apply for such a patent?

<u>Each author should answer the following 4 questions regarding disclosures not directly related to the current work:</u>

6. Do you have any other potential financial conflict of interest?

7. Do you have any affiliation with any commercial entity or organization not related to this particular study, but related to orthopaedic surgery or another branch of medicine?

8. Do you have any non-reimbursed relationships that may be seen as a conflict of interest?

9. Does your spouse, parent, child, or sibling have a relationship as described above in questions 1 through 8?

**Any payments to authors over $500 appearing in the Open Payments Database (https://openpaymentsdata.cms.gov) for the last 5 years should be included on this form. When disclosing potential conflicts, you do not need to provide dollar amounts, but please be sure to indicate the company and nature of payment: educational support, royalties, hospitality payments (food and lodging), etc.**

If none of the authors have answered, "Yes," to any of the above 9 questions, please mark option number 1 below, "No Potential Conflicts of Interest declared."

If one or more authors have answered, "Yes," to any of these 9 questions, please mark option number 2 below.

__ 1. Please insert "I (we) declare that we have no conflicts of interest in the authorship or publication of this contribution."

Or

_X_ 2. Please insert "One or more of the authors has declared a potential conflict of interest as specified in the AJSM Conflict of Interest statement."

Please specify below

- Which of the potential conflicts are being declared
- The specific author(s) declaring a potential conflict of interest
- Any other circumstance, which is not covered in the potential conflicts of interest listed above, that you feel might be perceived as constituting a potential conflict of interest and wish to voluntarily declare

**Albright**: Arthrex, Inc. /Gemini – Education and travel, speaking hospitality; Smith & Nephew - Research support, Wardlow Int – honoraria for speaking. **Algan**: Pylant Medical and Smith & Nephew provide educational support with occasional food to residency for training. They list me as a recipient although I do not receive any of the listed financial support. **Bowen:** Zimmer Biomet Holdings – Travel expenses **Crepeau:** Fortis Surgical – Support for education; Arthrex – Compensation for services other than consulting. **Edmonds:** Arthrex – Compensation for services other that consulting. **Ellington:** Medinc of Texas – Support for education. **Ellis**: Smith and Nephew – Education; Arthrex / Pylant Medical- Education; Smith & Nephew, Inc. - Travel/Lodging, Services other than consulting (faculty or speaker at a venue other than continuing education), Food and Beverage; Orthopaediatrics – Speaking; Pediatric Research in Sports Medicine (PRiSM) – Board member / Committee member; Pediatric Orthopaedic Society of North America (POSNA) – Committee member; American Orthopaedic Society of Sports Medicine (AOSSM) – Texas Delegate **Fabricant**: WishBone Medical – consultant. **Ganley**: American Journal of Sports Medicine – Associate Editor; AlloSource – Research Support for an unrelated project;

Arthrex – Educational Support; Vericel Corporation – Research Support for an unrelated project; Liberty Surgical, Inc – Educational Support; POSNA – Committee member; PRISM – Committee member; IPOS – Board or committee member; AAOS – Board or committee member; AAP Section on Orthopedics – Board member; Theodore Ganley has received research support from Allosource and Vericel and education payments from Arthrex and Liberty Surgical. **Green:** AAOS – Board or committee member; AO Trauma International – Paid presenter/speaker; Arthrex, Inc: IP Royalties, paid consultant, paid presenter or speaker; Current Opinion in Pediatrics – Editorial or governing board, publishing royalties, financial or material support; New York County Medical Society – Board or committee member; NY State Society of Orthopedic Surgeons – Board or Committee member; PatelloFemoral Foundation – Board or committee member; Pediatric Orthopaedic Society of North America – Board or committee member; Pediatric Research in Sports Medicine – Board or committee member; PEGA Medical – IP Royalties; Wolters Kluwer Health – Lippincott Williams & Wilkins – Publishing royalties, financial or material support; Synthes GmbH – Compensation for serving as faculty or as a speaker **Gupta**: Educational Conference/Cadaveric Labs and travel expenses with Arthrex, Stryker, and Wright Medical. **Heyworth:** Imagen Technologies, Inc – Stock owner and consultant; Arthrex, Inc. and Kairos Surgical – Educational Consultant, educational fees; Springer Science & Business Media – Royalties (textbook related); Pediatric Research in Sports Medicine (PRiSM) – Previous Board of Directors member (Director-at-Large); Pediatric Orthopaedic Society of North America (POSNA) – Committee member, committee chair. **Mayer:** Allosource - Consulting fees; Stryker Corp –Consulting fees; Smith and Nephew – Travel expenses; Gemini Mountain Medical – Support for education; Arthrex – Compensation for services other than consulting. **Milewski:** Elsevier, Inc - editorial royalties (unrelated to submission); Pediatric Research in Sports Medicine (PRiSM) Society – Board of Directors; Kairos Surgical – Support for education. **Niu:** Supreme Orthopedic Systems – Support for education; Arthrex – Support for education; Smith and Nephew – Travel expenses **Pacicca:** Titan Surgical Group – Support for education. **Parikh:** CDC Medical – Support for education. **Saper:** Arthrex, Inc. –Educational support, hospitality payments, compensation for services other than consulting; Summit Surgical Corp – Education support; Smith & Nephew – Educational support; DJO – Grant, hospitality payments. **Schmale:** Arthrex – Research support not related to this study; Summit Surgical Corp – Support for education; OrthoPediatrics – General payment; SIGN Fracture Care International – Compensation for services other than consulting. **Schmitz:** Elsevier Publishing – Royalties; HitCheck – stock options; JBJS Miller Review – Paid Speaker; Medinc of Texas – Travel and lodging, food and beverage; Stryker Corporation – Food and Beverage; Arthrex, Inc. – Education. **Shea:** Evolution Surgical – Support for education, food and beverage; Arthrex – Travel/lodging, food and beverage; BioMarin Pharmaceuticals: food and beverage; Sarcio Inc. – Medical advisory board member; nView, Inc. – Medical advisory board member; Medeloop, Inc. – Medical advisory board member. **Rhodes**: Consultant for OrthoPaediatrics ; research grant from Smith & Nephew with no financial ties to this study. **Wilson:** Pylant Medical / Arthrex – educational support

## Statement of Sponsorship

Please describe the sponsorship of your study, including any direct or indirect financial support and any donations of equipment, services, supplies, specimens, use of testing facilities, etc.

________________________________________

________________________________________

**Statement of Authorship**

All authors have contributed substantially to the conception, design, analysis, and/or interpretation of the data in this manuscript and will take public responsibility for the content.

___HBE____ Initials of corresponding author

Has anyone not listed as an author on this paper contributed to the conduct or analysis of this study or the editing, writing, or translation of this manuscript?

_____ Yes

___X__ No

If yes, please identify the individual(s) or entity and describe their role. Also, please include disclosure for these individuals.

**Description of Publication Restrictions**

Have all authors had unrestricted access to all the data of this study?

__X___Yes

______ No

If No, please describe the restrictions.

Have the named authors had the final authority over the content of this paper?

__X__Yes

_____No

If No, please identify the party who had the final authority.

**Statement of Related Publications**

Manuscripts must not have been published elsewhere in any language or substantially overlap or duplicate published work or work that is currently in preparation. Manuscripts must not be under simultaneous consideration by any other publication, before or during the peer-review process. Manuscripts should cite any other work by one or more of the co-authors that is relevant to the subject matter of the current submission or that used any of the same subjects, animals, or specimens being reported in the current submission. This includes manuscripts that are currently under preparation, are being considered by journals, are accepted for publication, or already published. In any of these cases, the relationship to the current submission should be made clear.

Do any authors have a related or similar publication in preparation, in press, or published?

__X___ Yes

_____ No

If yes, has this paper been referenced in the work submitted for consideration?

____ Yes

_____ No

If No, please cite or describe these works and explain the reasons for not citing them.

AJSM does not allow for publication of manuscripts already uploaded to preprint servers, and this would therefore disqualify the material from further consideration. Has this paper been uploaded to a preprint server?

_____ Yes

__X___ No

Signature of Corresponding Author (on behalf of all authors) ______________________

Title of paper: Surgical Characteristics of Pediatric and Adolescent Discoid Lateral Meniscus - Age Predicts Pathology and Treatment: A Report from the (XXXX) database ________

MS Number: __ AMJSPORTS/2022/336975 _______

Date: __8/28/2023____________
